# Update on novel pharmacological therapies for osteoarthritis

**DOI:** 10.1177/1759720X19864492

**Published:** 2019-07-23

**Authors:** Asim Ghouri, Philip G. Conaghan

**Affiliations:** Leeds Institute of Rheumatic and Musculoskeletal Medicine, University of Leeds and NIHR Leeds Biomedical Research Centre, Leeds, UK; Leeds Institute of Rheumatic and Musculoskeletal Medicine, University of Leeds and NIHR Leeds Biomedical Research Centre, Chapel Allerton Hospital, Chapeltown Rd, Leeds LS7 4SA, UK

**Keywords:** cartilage, corticosteroids, DMOAD, inflammation, nociceptive pain, osteoarthritis, synovitis

## Abstract

Osteoarthritis (OA) is a chronic painful arthritis with increasing global prevalence. Current management involves non-pharmacological interventions and commonly used pharmacological treatments that generally have limited analgesic efficacy and multiple side effects. New treatments are therefore required to relieve patient symptoms and disease impact. A number of existing pharmacological therapies have been recently trialled in OA. These include extended-release triamcinolone and conventional disease-modifying anti-rheumatic drugs (DMARDs) used in the management of rheumatoid arthritis; generally, DMARDs have not shown a benefit in treating OA. Novel analgesic therapies are in development, including those targeting peripheral pain pathways. Disease-modifying osteoarthritis drugs (DMOADs) target key tissues in the OA pathophysiology process and aim to prevent structural progression; a number of putative DMOADs are in phase II development. There is preliminary evidence of structural improvement with some of these therapies but without concomitant symptom improvement, raising new considerations for future DMOAD trials.

## Introduction

Osteoarthritis (OA) is a chronic, painful arthritis that can lead to reduced functioning and substantial disability in affected individuals. It is estimated that 242 million people worldwide are affected with OA of the hip or knee.^1^ The prevalence is increasing with an ageing population, along with rising risk factors such as obesity.^2^ The pathogenesis of OA is complex and is thought to develop as a result of the interplay between mechanical, genetic, metabolic and inflammatory mechanisms.^3^

Management of OA symptoms involves pharmacological and non-pharmacological strategies, with joint replacement considered in symptom-refractory disease. Analgesia remains the mainstay of pharmacological treatment for symptomatic OA, including paracetamol, topical and oral nonsteroidal anti-inflammatory drugs (NSAIDs) and opioid medications. However, benefits from paracetamol and opioids are limited, and NSAIDs and opioids are not suitable for many patients given their side-effect profile.^4^ Intra-articular therapies such as corticosteroids are also commonly used, though often with short-term benefits.

Given the central role of pain in the clinical syndrome of OA, new therapeutics have targeted peripheral nociceptive pain pathways. Disease-modifying osteoarthritis drugs (DMOADs) are a putative class of agents which aim to act on the key tissues involved in OA to prevent structural progression and therefore improve symptoms. Currently, no DMOADs have been licensed for use but a number of potential therapies are under investigation.

This narrative review will provide an overview for clinicians of recent advances in knowledge on the use of existing pharmacological therapies and discuss novel treatments currently under an advanced stage of investigation (at least in phase II trials) for the treatment of OA. The therapies discussed have primarily been investigated for the treatment of knee OA, with some having been trialled in hand OA.

### Scope of this review

Although this was a narrative review, we based it on a *PubMed* search for publications and a review of relevant meeting abstracts on pharmacotherapy trials in OA reported in 2017 and 2018. Only pharmacological therapies in at least phase II development for primary OA were selected; exercise, therapies marketed as devices (such as hyaluronans), nutraceuticals (such as glucosamine) and other non-pharmacological interventions were not included. Where relevant, older studies were referenced to give background detail on the candidate therapy.

### Update on existing therapies

#### Colchicine

While colchicine is not currently recommended for treatment of OA, it is commonly used for the treatment of gout and pseudogout. Basic calcium phosphate (BCP) crystals have been detected in synovial fluid in OA, with hydroxyapatite the most common form found in OA joints (detected in the cartilage of up to 100% of affected joints at the time of joint replacement).^5^ Positive correlations have been reported between synovial fluid BCP crystal levels and radiographic OA severity.^6^ BCP crystals activate the inflammasome including NOD-, LRR- and pyrin domain-containing 3 (NLRP3) which increase interleukin (IL)-1β expression, the levels of which also correlate with OA severity.^7,8^ Colchicine was recently trialled in OA as it is thought to block IL-1β release by inhibiting NLRP3.^9^ The three previous small trials found symptomatic improvement with colchicine in OA knee patients.^10–12^ A more recent double-blind, placebo-controlled, randomized trial compared colchicine 500 µg twice daily with placebo over 16 weeks in 109 patients with knee OA. The study did not achieve its primary endpoint of a significant improvement in Western Ontario and McMaster Universities Osteoarthritis Index (WOMAC) score at week 16.^13^ Although colchicine is therefore unlikely to provide a new OA therapy, understanding the place of treating nonurate crystal disease requires future consideration.

#### Hydroxychloroquine

Hydroxychloroquine has been used in clinical practice in patients with inflammatory hand OA with anecdotal evidence of benefit, in part because of its effectiveness in treating rheumatoid arthritis (RA) synovitis and an acceptable safety profile.^14,15^ It has a variety of immunomodulatory effects and was considered to potentially treat OA due to its inhibitory action on Toll-like receptor (TLR) signalling,^16^ as TLRs are upregulated in OA cartilage and thought to stimulate cartilage breakdown *via* proinflammatory pathways.^17,18^ In addition, there is evidence of synovitis in hand OA.^19,20^ Previous small pilot studies suggested improvements in symptoms after hydroxychloroquine treatment.^21,22^ A large randomized, double-blind, placebo-controlled clinical trial analysed 248 patients over a 12 month period.^23^ Patients with moderate to severe hand pain were randomized to hydroxychloroquine or placebo, in addition to their usual analgesic medication. The study did not demonstrate a significant reduction in hand pain with additional hydroxychloroquine compared with placebo at 6 months, thereby not achieving primary endpoint. There was also no significant difference in radiographic progression between treatment groups at 12 months. In a subset of patients, stratification for (commonly found) ultrasound-detected synovitis did not change the study results. Another randomized controlled trial comparing hydroxychloroquine 400 mg with placebo in 196 patients with hand OA (and not on concomitant NSAID or corticosteroid treatment) also did not detect a significant difference in pain scores after 24 weeks of treatment.^24^

#### Tumour necrosis factor inhibitors

There is evidence that tumour necrosis factor alpha (TNF-α) is implicated in OA pathogenesis;^25^ however, previous studies have not demonstrated adalimumab to be effective compared with placebo in reducing symptoms in hand OA.^26,27^ More recently, the HUMOR trial compared subcutaneous adalimumab 40 mg every other week with placebo over 12 weeks in a crossover trial of patients with erosive hand OA and evidence of magnetic resonance imaging (MRI)-deﬁned synovitis.^28^ A total of 43 patients were randomized and there was an 8 week washout period before treatment groups crossed over. No significant difference was detected in visual analogue scale (VAS) scores for pain between the treatment groups. No significant differences were detected for any secondary outcomes including change in MRI-detected synovitis and bone marrow lesions.

Etanercept has recently been studied in a 1 year, double-blind, randomized, placebo-controlled, multicentre trial of 90 patients with symptomatic erosive inflammatory hand OA. The study did not achieve its primary endpoint of a significant improvement in VAS pain at 24 weeks with etanercept 50 mg weekly.^29^ In addition, no significant treatment reduction in ultrasonographic or MRI-detected synovitis was seen after 1 year. The study also detected a significant reduction in MRI-detected bone marrow lesions in the interphalangeal joints of one hand after 1 year with etanercept; however, this was in a very small subgroup (*n* = 10 in each treatment group).

#### Injectable corticosteroids

Intra-articular corticosteroids are commonly used for OA, with most trials focusing on the knee. While patients experience substantial improvement in pain scores, symptomatic improvement tends to be short-lived, with no associated benefit seen at 6 months.^30^ A Cochrane review of 27 trials found an association with small-to-moderate improvement in function at up to 6 weeks, but no improvement beyond this timeframe.^31^ It also found moderate-to-large heterogeneity between trials.

##### Intra-articular triamcinolone acetonide extended release

Given the short-lived benefits of corticosteroid, FX006, a preparation of triamcinolone acetonide extended release (TA-ER) produced using microsphere technology, was trialled in patients with knee OA, with the aim of giving prolonged benefits. A phase IIa randomized, double-blind, controlled, dose-finding trial compared TA-ER at doses 10 mg, 40 mg and 60 mg with immediate-release triamcinolone 40 mg in 228 patients with knee OA.^32^ Patients were followed up for 12 weeks after single intra-articular knee injection. The results demonstrated a significant improvement in mean daily pain intensity scores with TA-ER 40 mg *versus* immediate-release triamcinolone 40 mg at weeks 5–10. In addition, all WOMAC subscale scores were superior in TA-ER 40 mg compared with immediate-release triamcinolone at 8 weeks. TA-ER 10 mg and 60 mg were not reported to be significantly superior to immediate-release triamcinolone 40 mg. TA-ER reported similar frequencies of adverse events (AEs) to immediate-release triamcinolone.

A further phase IIb study compared TA-ER with placebo in 306 patients with knee OA. The study failed to achieve its primary outcome of a significant improvement in average daily pain (ADP) intensity *versus* placebo at time point of 12 weeks; however, there were significant improvements in ADP intensity scores with TA-ER 32 mg *versus* placebo at weeks 1–11 and at week 13.^33^ A more recent phase III, multicentre, double-blinded, randomized, controlled trial compared TA-ER (32 mg) with immediate-release triamcinolone (40 mg) and placebo in 484 patients with knee OA.^34^ It achieved its primary endpoint of a significant improvement in ADP intensity compared with placebo at 12 weeks. There was no significant improvement in ADP intensity with TA-ER *versus* immediate-release triamcinolone at 12 weeks; however, the WOMAC pain, stiffness and physical function scores, and the Knee injury and Osteoarthritis Outcome Score (Quality of Life subdomain; KOOS-QoL) at 12 weeks significantly improved with TA-ER 32 mg compared with both placebo and immediate-release triamcinolone. The differences between the active comparator seen for the different outcome measures may well reflect a greater responsiveness for the disease-specific, multi-item WOMAC tool over the ADP single item question. Given the significant improvement over placebo, TA-ER has been licensed by the United States Food and Drug Administration (US FDA) for managing OA-related knee pain. One further advantage of TA-ER’s mechanism of action with slow intra-articular release, is reduced systemic exposure compared with immediate-release triamcinolone.^35^ It has been demonstrated that TA-ER 32 mg gives less glycaemic disruption compared with standard triamcinolone 40 mg in patients with type 2 diabetes.^36^

##### Intramuscular corticosteroid

While intra-articular corticosteroid injections are of short-term benefit in hip OA,^37^ a recent randomized, double-blind, trial compared intramuscular triamcinolone acetate 40 mg with placebo in patients with hip OA.^38^ Intramuscular injections require less training and would be of potential benefit in primary care management of OA. Pain levels at rest and on walking using an 11-point numeric rating scale (NRS; 0–10, where 0 = no pain) and WOMAC pain levels were recorded at 2, 4, 6 and 12 weeks after injection. The results from 106 patients were analysed and demonstrated a significant reduction in NRS hip pain at rest in the triamcinolone group compared with the placebo control group at 2 weeks. This significant difference persisted for the whole 12 weeks of the trial period. No significant difference in pain on walking and WOMAC pain was demonstrated at 2 weeks. However, triamcinolone was significantly superior to placebo at reducing pain on walking at 4, 6 and 12 weeks. Triamcinolone was also significantly superior in reducing WOMAC pain, function, stiffness and total scores compared with placebo at weeks 4, 6 and 12. The magnitude of improvement in NRS pain at 2 weeks was reported as probably clinically relevant, but not for beyond that time point. In addition, only one of the three primary outcome measurements were achieved at 2 weeks.

#### Bisphosphonates

Subchondral bone pathology is integral to the OA process and a number of therapies used for osteoporosis have been explored as OA therapies. Strontium ranelate was studied in the SEKOIA trial, a randomized, double-blind, 3-year study involving 1683 patients with symptomatic primary knee OA and compared strontium 1 or 2 g/day with placebo.^39^ The study demonstrated a significant reduction in joint space width degradation with both strontium doses compared with placebo. Results also showed a significant improvement in WOMAC total score and pain subscore at 2 g/day; however, a 14% annualized dropout rate highlights the complex issues of participant retention in long-term OA trials and handling missing data.

Bisphosphonates, related to their anti-osteoclastic actions, have also been trialled in OA, where their mechanism of action may have benefits on subchondral bone and cartilage. A meta-analysis of seven randomized, placebo-controlled trials did not show symptomatic improvement or reduction in radiographic OA progression with bisphosphonate therapy.^40^ The analysis did suggest bisphosphonates may still have potential benefit in a subset of patients with high rates of subchondral bone turnover. Recent studies have recruited patients with subchondral bone abnormalities [patients with bone marrow lesions (BMLs)]. BMLs are commonly seen on MRI scans of OA knees and are associated with both pain and progressive cartilage loss.^41–44^ A double-blind, parallel-group, placebo-controlled trial of zoledronic acid (ZA) recruited 59 patients aged 50–80 years with knee pain and at least one BML on MRI.^45^ Significant symptomatic benefit and a reduction in BMLs were reported at 6 months. The preliminary report from the larger follow-up, multicentre, randomized controlled trial, comparing once-yearly intravenous infusion of ZA 5 mg with placebo on knee pain and BML size over 24 months in 223 patients with knee OA, significant knee pain and MRI-detected BMLs has recently been presented.^46^ However in this new trial, no significant difference in WOMAC pain score, WOMAC function score, or BML change was detected between treatment groups after 24 months.

### Therapies in advanced trial development

#### Intra-articular capsaicin

Transient receptor potential cation channel subfamily V member 1 (TRPV1) is a protein expressed on nociceptive nerve fibres (Aδ and C) and its activation leads to a prolonged refractory state known as desensitization^47^ It is therefore an attractive target for potential analgesic medication. CNTX-4975 is a highly purified, synthetic trans-capsaicin with specific activity for TRPV1-containing pain nociceptors. It does not impact other sensory fibres such as touch or pressure and is the first intra-articular capsaicin preparation.^48^ Previous evidence has supported topical capsaicin in relieving OA pain.^49–52^ A 24-week, randomized, double-blind, placebo-controlled, dose-ranging study demonstrated significant improvement in WOMAC A1 pain score (defined as how much pain a patient has when walking on a flat surface) at 12 and 24 weeks with a single dose CNTX-4975 1 mg knee injection in patients with moderately painful knee OA.^53^ Data on AEs are limited currently but the most common treatment-emergent adverse event is reported to be arthralgia.^53^ A phase III trial and a study examining efficacy of repeated doses of CNTX-4975 are currently in progress.^54,55^

#### Anti-nerve growth factor monoclonal antibodies

Nerve growth factor (NGF) is a neurotrophin with increased expression in OA and is thought to stimulate growth of nociceptive nerve fibres and expression of nociceptive cell surface receptors.^56^ The joint capsule, ligaments, periosteum, menisci, subchondral bone and synovium are all highly innervated with nociceptive nerve fibres and potential sources of joint and knee pain in OA.^57^ The peripheral nociceptive pathway therefore offers novel targets for analgesic agents.

Tanezumab is a monoclonal antibody strongly targeting NGF, thereby preventing it from binding its receptor with the overall aim of reducing pain.^58^ Other anti-NGF biologics on trial include fasinumab and fulranumab, although development of fulranumab has now terminated. A meta-analysis of 9 studies with 10 randomized controlled trials enrolling 7665 patients compared tanezumab with placebo/active comparator in knee or hip OA. This demonstrated superiority in efficacy (WOMAC pain subscale, WOMAC function subscale, patient global assessment) with tanezumab.^59^ Older studies investigated intravenous (IV) tanezumab but more recent studies have used subcutaneous (SC) preparations. In the phase III trials, fixed doses of tanezumab were used (2.5 mg, 5 mg and 10 mg). A recent presentation of a phase III trial compared fixed doses 8 weeks apart of SC tanezumab *versus* step-up dosing (2.5 mg administered at baseline, 5 mg administered at week 8) in 696 patients with hip/knee OA who had not responded to, or were unable to tolerate, standard pain treatments. These preliminary data demonstrated tanezumab 2.5 mg was superior to placebo in improving WOMAC pain, WOMAC function and patient global assessment scores at week 16. Further benefit was demonstrated by increasing the dose of tanezumab from 2.5 mg to 5 mg at week 8.^60^

Tanezumab has also been compared with NSAIDs (celecoxib 100 mg and naproxen 500 mg) and oxycodone 10–40 mg, with tanezumab monotherapy at 5 mg and 10 mg demonstrating superior efficacy to each of these drugs.^61,62^ Combined tanezumab and NSAID therapy also demonstrated superior analgesic efficacy compared with NSAID monotherapy but not tanezumab monotherapy.^62^

A number of AEs have been reported with tanezumab, although rates of discontinuation due to these are low.^59^ Tanezumab-treated patients are significantly more likely to experience paraesthesia, headaches, arthralgia, peripheral oedema, peripheral neuropathy, hypo- and hyper-aesthesia. Arthralgia was the most commonly reported side effect (8% of tanezumab-treated patients). Lower doses of tanezumab are associated with fewer AEs.^59^

Rapidly progressive OA (RPOA) is the most serious AE reported with tanezumab and fulranumab, with the risk of RPOA being dose-responsive.^63^ It is a painful condition diagnosed radiographically by rapid joint space narrowing and severe progressive atrophic bone and has been reported in 1% of patients who received tanezumab.^64^ In recent trials, tanezumab is used at a maximal 5 mg dose in patients with hip or knee OA as the risk appears lower and is outweighed by its potential therapeutic benefit.^65^ The combination of tanezumab and NSAIDs appears to increase the risk of RPOA compared with tanezumab alone.^65^

### Potential DMOADs

It should be possible to modify key structures or tissues within the OA joint; however, the challenges for DMOAD trials include: the very slow rate of cartilage loss in OA knees over time, the insensitivity of radiographic outcome measures resulting in the need for very large trials, and poor understanding of the relationship between structure and symptoms.

#### Sprifermin

Sprifermin (rhFGF18) is recombinant fibroblast growth factor 18 administered *via* intra-articular route and acts on FGFR3 receptors in cartilage.^66^ Animal models of OA have shown that fibroblast growth factor 18 can stimulate cartilage growth by proliferation of chondrocytes and modulating extracellular matrix turnover.^67,68^

A 1 year, randomized, double-blind, placebo-controlled, proof-of-concept trial compared sprifermin doses of 10 μg, 30 μg, and 100 μg.^69^ Patients were given two cycles of three once-weekly injections (weeks 0–2 and 13–15) of sprifermin or placebo. Of the 168 patients evaluated, sprifermin did not achieve its primary efficacy endpoint of significant reduction in central medial femorotibial compartment cartilage thickness loss at 6 or 12 months; however, sprifermin (at all doses) did achieve its prespecified secondary structural efficacy MRI endpoints, including a significant reductions in the loss of total femorotibial and lateral femorotibial cartilage thickness and reduced loss of radiographically evident lateral joint space width at 12 months.

A 5-year phase II, dose-ranging, randomized, placebo-controlled trial (FORWARD) is currently in progress. A total of 549 patients were randomized of which 18.4% (sprifermin) and 24.1% (placebo) discontinued the study within 3 years. Structural endpoints (cartilage thickness) were measured by quantitative MRI at the tibiofemoral joint. Preliminary results at 3 years demonstrate the expected (natural history) reduction in mean cartilage thickness from baseline to 3 years was significantly lower in patients on sprifermin 100 μg than placebo at the total femorotibial joint, and in the medial, lateral, central medial and central-lateral subregion. There was an initial increase in overall cartilage thickness with sprifermin 100 μg for the first 2 years; however, overall cartilage thickness reduced for both sprifermin 100 μg and placebo groups between years 2 and 3 but the significant difference between treatment groups was maintained.^70^ Of note, no difference in symptom improvement was reported between treatment groups. This raises an important issue for potentially effective treatments improving OA structural pathologies: how long after changing structure will we need to follow patients in order to see a difference in patient important outcomes (such as reduction in symptoms or joint replacement)?

#### Wnt pathway inhibition

SM04690 is an inhibitor of the Wnt signalling pathway, a signal transduction pathway thought to play a role in cartilage degeneration and the pathogenesis of OA, through their effects on chondrocyte, osteoblast and synovial cell differentiation.^71,72^ Alterations in genes encoding Wnt signalling pathway proteins have been detected in murine and human OA tissues, along with lower levels of the Wnt inhibitory protein DKK1.^72^ A phase II randomized, double-blind, placebo-controlled trial comparing three doses of SM04690 (low, medium and high doses) in 455 patients with knee OA has recently been completed. A prespecified subpopulation of this group with unilateral symptomatic knee OA (*n* = 164) was also analysed, based on the hypothesis that patients with symptomatic OA in both knees may not respond as well as those with only one knee affected (a ‘widespread pain’ interference phenomenon). A further subpopulation, patients with unilateral knee OA without widespread pain (*n* = 128), was also analysed. Results have shown that in the intention-to-treat population, there was no significant difference in WOMAC A1 (pain on walking) improvement between treatment groups; however, patients receiving the medium dose (0.07 mg) SM04690 had significant improvement in their WOMAC A1 score compared with placebo in unilateral symptomatic knee patients at 39 and 52 weeks and unilateral symptomatic patients without widespread pain at 26, 39 and 52 weeks.^73^

#### Interleukin-1α and β inhibition

In OA, IL-1α and IL-1β are expressed in increased levels within the cartilage and synovial membrane.^74,75^ Elevated IL-1 levels are associated with increased expression of markers of OA pathophysiology including catabolic enzymes, prostaglandins, nitric oxide and other markers in OA fluids and tissue.^76^ In addition, blockage of the IL-1 receptor can slow the progression of OA in animal models.^77–80^

Anakinra is a recombinant form of an IL-1 receptor antagonist (IL-1Ra). A multicentre, double-blind, placebo-controlled study randomized 170 patients to receive a single intra-articular injection of placebo, anakinra 50 mg, or anakinra 150 mg in their symptomatic knee. Although anakinra was well tolerated, no significant difference in the mean WOMAC pain score improvements from baseline to week 4 could be detected between the treatment groups.^81^

Lutikizumab (formerly ABT-981) is a novel human dual variable domain immunoglobulin that binds and inhibits the actions of IL-1α and IL-1β.^82^ A randomized, double-blind, placebo-controlled, parallel-group phase II trial (ILLUSTRATE-K) compared two-weekly SC injections of lutikizumab at 25 mg, 100 mg, or 200 mg in patients with knee OA for 50 weeks.^83^ Preliminary results demonstrated significant improvement in WOMAC pain score at 16 weeks with lutikizumab 100 mg compared with placebo (achieving the primary endpoint); however, no significant improvement compared with placebo was demonstrated with lutikizumab 25 mg or 200 mg at 16 weeks. Cartilage thickness, MRI synovitis, and other structural endpoints were similar between lutikizumab and placebo, although lutikizumab was generally well tolerated. Given the lack of dose response and failure to meet structural endpoints, there is uncertainty regarding the clinical efficacy of lutikizumab from this trial.

Lutikizumab has also been trialled in patients with erosive hand OA. A phase IIa, placebo-controlled, randomized study measured clinical and radiological outcomes in 131 patients with hand OA as per American College of Rheumatology criteria (⩾3 inflamed interphalangeal joints which are tender, swollen, or both, hand pain ⩾6 (scale 0–10), and ⩾1 erosive interphalangeal joint on X-ray). Patients were given lutikizumab 200 mg (*n* = 67) or placebo (*n* = 64) every 2 weeks for 26 weeks. Preliminary data did not demonstrate a significant difference in Australian/Canadian Hand OA Index (AUSCAN) pain improvement scores between treatment groups at 16 weeks. In addition, there was no significant difference in X-Ray or MRI data between treatment groups.^84^

The IL-1 story continues to be intriguing. Canakinumab is a monoclonal antibody targeting IL-1β. Previous *in vitro* work on human chondrocytes demonstrated increased proteoglycan and reduced nitric oxide synthesis which may result in reduced cartilage breakdown.^85^ A recent very large randomized, placebo-controlled trial investigated the cardiovascular effect of subcutaneous canakinumab at doses 50 mg, 150 mg or 300 mg every 3 months for a median of 3.7 years, involving 10,061 patients with previous myocardial infarction and a high-sensitivity C-reactive protein level ⩾2 mg/l on blood testing.^86^ The results demonstrated canakinumab at a dose of 150 mg was associated with a significantly lower rate of recurrent cardiovascular events compared with placebo, independent of lipid-level lowering. Although this was primarily a cardiovascular study, a substudy of this trial has recently reported there is also a reduced incidence of OA symptoms and total knee and hip replacements in the patients who received canakinumab.^87^

#### Cathepsin K inhibition

Cathepsin K is a cysteine protease involved in bone resorption, degrading types I and II collagen and aggrecan found in cartilage. MIV-711 is a potent, selective and reversible inhibitor of cathepsin K which aims to prevent degradation of cartilage, thereby improving symptoms in OA. A 6-month, multicentre, randomized, placebo-controlled, double-blind, three-arm parallel, phase IIa study evaluating the efficacy, safety and tolerability of MIV-711 in patients with knee OA was completed in 2017 and preliminary results presented.^88^ Patients with knee OA received MIV-711 100 mg, 200 mg or matched placebo four times daily for 26 weeks. A significant reduction in pain and QoL scores was not detected, although there was a trend towards a reduction in pain scores with 100 mg and 200 mg doses at 26 weeks. However, a unique aspect of this trial was the key secondary outcome of three-dimensional quantitative bone area and this novel imaging biomarker can potentially be used as an outcome measure in further DMOAD trials.^89^ The results demonstrated a significant reduction in femoral OA bone disease progression on MRI at week 26 for MIV-711 100 mg and 200 mg doses *versus* placebo. There was also a significant reduction in the loss of cartilage thickness on the medial femur for 100 mg *versus* placebo. The main AEs reported were musculoskeletal symptoms, skin disorders and infections, but there was a reported overall acceptable safety profile.^90^ Further studies are needed to evaluate the potential structure-modifying effects of this agent.

## Conclusion

Current treatment options in OA remain limited. Conventional RA DMARDs have not so far demonstrated benefit in managing OA symptoms; more data on methotrexate are expected soon;^91^ however, recent trials involving peripheral nociceptive targets have demonstrated promising analgesic results in knee OA. The two recent studies of both a cartilage anabolic agent and an osteoclast inhibitor suggest a potential for structure modification but without symptom benefit; this may be too hard to achieve in a typical duration OA trial. Much further thought is needed on what a ‘successful’ DMOAD trial will look like going forward.

## References

[1] Global Burden of Disease Study 2013 Collaborators. Global, regional, and national incidence, prevalence, and years lived with disability for 301 acute and chronic diseases and injuries in 188 countries, 1990–2013: a systematic analysis for the global burden of disease study 2013. Lancet 2015; 386: 743–800. 26063472 10.1016/S0140-6736(15)60692-4 PMC4561509

[2] Neogi T Zhang Y. Epidemiology of osteoarthritis. Rheum Dis Clin North Am 2013; 39: 1–19. 23312408 10.1016/j.rdc.2012.10.004 PMC3545412

[3] Chen D Shen J Zhao W et al Osteoarthritis: toward a comprehensive understanding of pathological mechanism. Bone Res 2017; 5: 16044. 28149655 10.1038/boneres.2016.44 PMC5240031

[4] Zhang W Nuki G Moskowitz RW et al OARSI recommendations for the management of hip and knee osteoarthritis: part III: changes in evidence following systematic cumulative update of research published through January 2009. Osteoarthritis Cartilage 2010; 18: 476–499. 20170770 10.1016/j.joca.2010.01.013

[5] Fuerst M Bertrand J Lammers L et al Calcification of articular cartilage in human osteoarthritis. Arthritis Rheum 2009; 60: 2694–2703. 19714647 10.1002/art.24774

[6] Halverson PB McCarty DJ. Patterns of radiographic abnormalities associated with basic calcium phosphate and calcium pyrophosphate dihydrate crystal deposition in the knee. Ann Rheum Dis 1986; 45: 603. 3017246 10.1136/ard.45.7.603 PMC1001944

[7] Denoble AE Huffman KM Stabler TV et al Uric acid is a danger signal of increasing risk for osteoarthritis through inflammasome activation. Proc Natl Acad Sci U S A 2011; 108: 2088. 21245324 10.1073/pnas.1012743108 PMC3033282

[8] McCarthy GM Dunne A. Calcium crystal deposition diseases - beyond gout. Nat Rev Rheumatol 2018; 14: 592–602. 30190520 10.1038/s41584-018-0078-5

[9] Leung YY Yao Hui LL Kraus VB. Colchicine—update on mechanisms of action and therapeutic uses. Semin Arthritis Rheum 2015; 45: 341–350. 26228647 10.1016/j.semarthrit.2015.06.013 PMC4656054

[10] Das SK Ramakrishnan S Mishra K et al A randomized controlled trial to evaluate the slow-acting symptom-modifying effects of colchicine in osteoarthritis of the knee: a preliminary report. Arthritis Rheum 2002; 47: 280–284. 12115158 10.1002/art.10455

[11] Das SK Mishra K Ramakrishnan S et al A randomized controlled trial to evaluate the slow-acting symptom modifying effects of a regimen containing colchicine in a subset of patients with osteoarthritis of the knee. Osteoarthritis Cartilage 2002; 10: 247–252. 11950246 10.1053/joca.2002.0516

[12] Aran S Malekzadeh S Seifirad S. A double-blind randomized controlled trial appraising the symptom-modifying effects of colchicine on osteoarthritis of the knee. Clin Exp Rheumatol 2011; 29: 513–518. 21640042

[13] Leung YY Haaland B Huebner JL et al Colchicine lack of effectiveness in symptom and inflammation modification in knee osteoarthritis (COLKOA): a randomized controlled trial. Osteoarthritis Cartilage 2018; 26: 631–640. 29426008 10.1016/j.joca.2018.01.026

[14] Haar D Sølvkjær M Unger B et al A double-blind comparative study of hydroxychloroquine and dapsone, alone and in combination, in rheumatoid arthritis. Scand J Rheumatol 1993; 22: 113–118. 8316771 10.3109/03009749309099254

[15] Clark P Casas E Tugwell P et al Hydroxychloroquine compared with placebo in rheumatoid arthritis: a randomized, controlled trial. Ann Intern Med 1993; 119: 1067–1071. 8239224 10.7326/0003-4819-119-11-199312010-00002

[16] Kyburz D Brentano F Gay S. Mode of action of hydroxychloroquine in RA—evidence of an inhibitory effect on toll-like receptor signaling. Nat Clin Pract Rheumatol 2006; 2: 458. 16951696 10.1038/ncprheum0292

[17] Kim HA Cho ML Choi HY et al The catabolic pathway mediated by Toll-like receptors in human osteoarthritic chondrocytes. Arthritis Rheum 2006; 54: 2152–2163. 16802353 10.1002/art.21951

[18] Sillat T Barreto G Clarijs P et al Toll-like receptors in human chondrocytes and osteoarthritic cartilage. Acta Orthop 2013; 84: 585–592. 24237425 10.3109/17453674.2013.854666 PMC3851674

[19] Keen HI Wakefield RJ Grainger AJ et al An ultrasonographic study of osteoarthritis of the hand: synovitis and its relationship to structural pathology and symptoms. Arthritis Rheum 2008; 59: 1756–1763. 19035429 10.1002/art.24312

[20] Vlychou M Koutroumpas A Malizos K et al Ultrasonographic evidence of inflammation is frequent in hands of patients with erosive osteoarthritis. Osteoarthritis Cartilage 2009; 17: 1283–1287. 19447214 10.1016/j.joca.2009.04.020

[21] Bryant LR des Rosier KF Carpenter MT. Hydroxychloroquine in the treatment of erosive osteoarthritis. J Rheumatol 1995; 22: 1527–1531. 7473478

[22] Punzi L Bertazzolo N Pianon M et al Soluble interleukin 2 receptors and treatment with hydroxychloroquine in erosive osteoarthritis. J Rheumatol 1996; 23: 1477–1478. 8856633

[23] Kingsbury SR Tharmanathan P Keding A et al Hydroxychloroquine effectiveness in reducing symptoms of hand osteoarthritis: a randomized trial. Ann Intern Med 2018; 168: 385–395. 29459986 10.7326/M17-1430

[24] Lee W Ruijgrok L Boxma-de Klerk B et al Efficacy of hydroxychloroquine in hand osteoarthritis: a randomized, double-blind, placebo-controlled trial. Arthritis Care Res (Hoboken) 2017; 70: 1320–1325. 10.1002/acr.23471 29125901

[25] Wojdasiewicz P Poniatowski Szukiewicz D. The role of inflammatory and anti-inflammatory cytokines in the pathogenesis of osteoarthritis. Mediators Inflamm 2014; 2014: 19. 10.1155/2014/561459 PMC4021678 24876674

[26] Verbruggen G Wittoek R Cruyssen BV et al Tumour necrosis factor blockade for the treatment of erosive osteoarthritis of the interphalangeal finger joints: a double blind, randomised trial on structure modification. Ann Rheum Dis 2012; 71: 891. 22128078 10.1136/ard.2011.149849 PMC3371224

[27] Chevalier X Ravaud P Maheu E et al Adalimumab in patients with hand osteoarthritis refractory to analgesics and NSAIDs: a randomised, multicentre, double-blind, placebo-controlled trial. Ann Rheum Dis 2015; 74: 1697–1705. 24817417 10.1136/annrheumdis-2014-205348

[28] Aitken D Laslett LL Pan F et al A randomised double-blind placebo-controlled crossover trial of HUMira (adalimumab) for erosive hand OsteoaRthritis – the HUMOR trial. Osteoarthritis Cartilage 2018; 26: 880–887. 29499287 10.1016/j.joca.2018.02.899

[29] Kloppenburg M Ramonda R Bobacz K et al Etanercept in patients with inflammatory hand osteoarthritis (EHOA): a multicentre, randomised, double-blind, placebo-controlled trial. Ann Rheum Dis 2018; 77: 1757. 30282670 10.1136/annrheumdis-2018-213202

[30] da Costa BR Hari R Jüni P. Intra-articular corticosteroids for osteoarthritis of the knee. JAMA 2016; 316: 2671–2672. 28027351 10.1001/jama.2016.17565

[31] Arroll B Goodyear-Smith F. Corticosteroid injections for osteoarthritis of the knee: meta-analysis. BMJ 2004; 328: 869. 15039276 10.1136/bmj.38039.573970.7C PMC387479

[32] Bodick N Lufkin J Willwerth C et al An intra-articular, extended-release formulation of triamcinolone acetonide prolongs and amplifies analgesic effect in patients with osteoarthritis of the knee: a randomized clinical trial. J Bone Joint Surg Am 2015; 97: 877–888. 26041848 10.2106/JBJS.N.00918

[33] Conaghan PG Cohen SB Berenbaum F et al Brief report: a phase IIb trial of a novel extended-release microsphere formulation of triamcinolone acetonide for intraarticular injection in knee osteoarthritis. Arthritis Rheumatol 2018; 70: 204–211. 29088579 10.1002/art.40364 PMC5814922

[34] Conaghan PG Hunter DJ Cohen SB et al Effects of a single intra-articular injection of a microsphere formulation of triamcinolone acetonide on knee osteoarthritis pain: a double-blinded, randomized, placebo-controlled, multinational study. JBJS 2018; 100: 666–677. 10.2106/JBJS.17.00154 PMC5916484 29664853

[35] Kraus VB Conaghan PG Aazami HA et al Synovial and systemic pharmacokinetics (PK) of triamcinolone acetonide (TA) following intra-articular (IA) injection of an extended-release microsphere-based formulation (FX006) or standard crystalline suspension in patients with knee osteoarthritis (OA). Osteoarthritis Cartilage 2018; 26: 34–42. 29024802 10.1016/j.joca.2017.10.003

[36] Conaghan PG Russell SJ Sala R et al Triamcinolone acetonide extended-release injectable suspension (TA-ER) is associated with reduced blood glucose elevation vs. standard triamcinolone in type 2 diabetes mellitus patients with knee osteoarthritis: a randomized, blinded, parallel-group study. Osteoarthritis Cartilage 2018; 26: S230.

[37] McCabe PS Maricar N Parkes MJ et al The efficacy of intra-articular steroids in hip osteoarthritis: a systematic review. Osteoarthritis Cartilage 2016; 24: 1509–1517. 27143362 10.1016/j.joca.2016.04.018

[38] Dorleijn DMJ Luijsterburg PAJ Reijman M et al Intramuscular glucocorticoid injection versus placebo injection in hip osteoarthritis: a 12-week blinded randomised controlled trial. Ann Rheum Dis 2018; 77: 875. 29514801 10.1136/annrheumdis-2017-212628

[39] Reginster JY Badurski J Bellamy N et al Efficacy and safety of strontium ranelate in the treatment of knee osteoarthritis: results of a double-blind, randomised placebo-controlled trial. Ann Rheum Dis 2013; 72: 179. 23117245 10.1136/annrheumdis-2012-202231 PMC3599139

[40] Vaysbrot EE Osani MC Musetti MC et al Are bisphosphonates efficacious in knee osteoarthritis? A meta-analysis of randomized controlled trials. Osteoarthritis Cartilage 2018; 26: 154–164. 29222056 10.1016/j.joca.2017.11.013

[41] Felson DT Chaisson CE Hill CL et al The association of bone marrow lesions with pain in knee osteoarthritis. Ann Intern Med 2001; 134: 541–549. 11281736 10.7326/0003-4819-134-7-200104030-00007

[42] Zhai G Blizzard L Srikanth V et al Correlates of knee pain in older adults: Tasmanian older adult cohort study. Arthritis Rheum 2006; 55: 264–271. 16583417 10.1002/art.21835

[43] Felson DT McLaughlin S Goggins J et al Bone marrow edema and its relation to progression of knee osteoarthritis. Ann Intern Med 2003; 139: 330–336. 12965941 10.7326/0003-4819-139-5_part_1-200309020-00008

[44] Castañeda S Roman-Blas JA Largo R et al Subchondral bone as a key target for osteoarthritis treatment. Biochem Pharmacol 2012; 83: 315–323. 21964345 10.1016/j.bcp.2011.09.018

[45] Laslett LL Doré DA Quinn SJ et al Zoledronic acid reduces knee pain and bone marrow lesions over 1 year: a randomised controlled trial. Ann Rheum Dis 2012; 71: 1322. 22355040 10.1136/annrheumdis-2011-200970

[46] Cai G Aitken D Laslett L et al OP0016 a multicentre randomised controlled trial of zoledronic acid for osteoarthritis of the knee with bone marrow lesions. Ann Rheum Dis 2018; 77: 57.

[47] Szallasi A Cortright DN Blum CA et al The vanilloid receptor TRPV1: 10 years from channel cloning to antagonist proof-of-concept. Nat Rev Drug Discov 2007; 6: 357. 17464295 10.1038/nrd2280

[48] Centrexion Therapeutics. A novel approach to treating pain. Boston, MA: Centrexion Therapeutics, 2018.

[49] Altman RD Aven A Holmburg CE et al Capsaicin cream 0.025% as monotherapy for osteoarthritis: a double-blind study. Semin Arthritis Rheum 1994; 23: 25–33.

[50] Mason L Moore RA Derry S et al Systematic review of topical capsaicin for the treatment of chronic pain. BMJ 2004; 328: 991. 15033881 10.1136/bmj.38042.506748.EE PMC404499

[51] Zhang WY Li-Wan-Po A. The effectiveness of topically applied capsaicin. A meta-analysis. Eur J Clin Pharmacol 1994; 46: 517–522. 7995318 10.1007/BF00196108

[52] Kosuwon W Sirichativapee W Wisanuyotin T et al Efficacy of symptomatic control of knee osteoarthritis with 0.0125% of capsaicin versus placebo. J Med Assoc Thai 2010; 93: 1188–1195. 20973322

[53] Stevens R Petersen D Ervin J et al OP0167 Efficacy and safety of CNTX-4975 in subjects with moderate to severe osteoarthritis knee pain: 24-week, randomized, double-blind, placebo-controlled, dose-ranging study. Ann Rheum Dis 2017; 76: 121.

[54] Stevens R A clinical study to test efficacy and safety of CNTX-4975-05 in patients with osteoarthritis knee pain. https://clinicaltrials.gov/ct2/show/NCT03429049?id=NCT02508155+OR+NCT03660943+OR+NCT03429049&rank=2&load=cart (accessed 10 September 2018)

[55] Stevens R A clinical study to test efficacy and safety of repeat doses of CNTX-4975-05 in patients with osteoarthritis knee pain. https://clinicaltrials.gov/ct2/show/NCT03660943?id=NCT02508155+OR+NCT03660943+OR+NCT03429049&rank=1&load=cart (accessed 10 September 2018)

[56] Denk F Bennett DL McMahon SB. Nerve growth factor and pain mechanisms. Annu Rev Neurosci 2017; 40: 307–325. 28441116 10.1146/annurev-neuro-072116-031121

[57] Malfait AM Schnitzer TJ. Towards a mechanism-based approach to pain management in osteoarthritis. Nat Rev Rheumatol 2013; 9: 654. 24045707 10.1038/nrrheum.2013.138 PMC4151882

[58] Abdiche YN Malashock DS Pons J. Probing the binding mechanism and affinity of tanezumab, a recombinant humanized anti-NGF monoclonal antibody, using a repertoire of biosensors. Protein Sci 2009; 17: 1326–1335. 10.1110/ps.035402.108 PMC2492818 18505735

[59] Chen J Li J Li R et al Efficacy and safety of tanezumab on osteoarthritis knee and hip pains: a meta-analysis of randomized controlled trials. Pain Med 2017; 18: 374–385. 28034979 10.1093/pm/pnw262

[60] Schnitzer TJ Easton R Pang S et al Efficacy and safety of subcutaneous tanezumab for the treatment of osteoarthritis of the hip or knee. Arthritis Rheumatol 2018; 70(Suppl. 10): abstract L20.

[61] Spierings EL Fidelholtz J Wolfram G et al A phase III placebo- and oxycodone-controlled study of tanezumab in adults with osteoarthritis pain of the hip or knee. Pain 2013; 154: 1603–1612. 23707270 10.1016/j.pain.2013.04.035

[62] Schnitzer TJ Ekman EF Spierings EL et al Efficacy and safety of tanezumab monotherapy or combined with non-steroidal anti-inflammatory drugs in the treatment of knee or hip osteoarthritis pain. Ann Rheum Dis 2015; 74: 1202. 24625625 10.1136/annrheumdis-2013-204905

[63] Hochberg MC. Serious joint-related adverse events in randomized controlled trials of anti-nerve growth factor monoclonal antibodies. Osteoarthritis Cartilage 2015; 23: S18–S21. 25527216 10.1016/j.joca.2014.10.005

[64] Hochberg MC Tive LA Abramson SB et al When is osteonecrosis not osteonecrosis? Adjudication of reported serious adverse joint events in the tanezumab clinical development program. Arthritis Rheumatol 2015; 68: 382–391. 10.1002/art.39492 26554876

[65] Miller RE Block JA Malfait AM. What is new in pain modification in osteoarthritis? Rheumatology (Oxford) 2018; 57: iv99–iv107. 29361112 10.1093/rheumatology/kex522 PMC5905627

[66] Davidson D Blanc A Filion D et al Fibroblast growth factor (FGF) 18 signals through FGF receptor 3 to promote chondrogenesis. J Biol Chem 2005; 280: 20509–20515. 15781473 10.1074/jbc.M410148200

[67] Moore EE Bendele AM Thompson DL et al Fibroblast growth factor-18 stimulates chondrogenesis and cartilage repair in a rat model of injury-induced osteoarthritis. Osteoarthritis Cartilage 2005; 13: 623–631. 15896984 10.1016/j.joca.2005.03.003

[68] Reker D Kjelgaard-Petersen CF Siebuhr AS et al Sprifermin (rhFGF18) modulates extracellular matrix turnover in cartilage explants ex vivo. J Transl Med 2017; 15: 250. 29233174 10.1186/s12967-017-1356-8 PMC5727954

[69] Lohmander LS Hellot S Dreher D et al Intraarticular sprifermin (recombinant human fibroblast growth factor 18) in knee osteoarthritis: a randomized, double-blind, placebo-controlled trial. Arthritis Rheumatol 2014; 66: 1820–1831. 24740822 10.1002/art.38614

[70] Hochberg M Guermazi A Guehring H et al OP0059 Efficacy and safety of intra-articular sprifermin in symptomatic radiographic knee osteoarthritis: pre-specified analysis of 3-year data from a 5-year randomised, placebo-controlled, phase II study. Ann Rheum Dis 2018; 77: 80–81.

[71] Deshmukh V Hu H Barroga C et al A small-molecule inhibitor of the Wnt pathway (SM04690) as a potential disease modifying agent for the treatment of osteoarthritis of the knee. Osteoarthritis Cartilage 2018; 26: 18–27. 28888902 10.1016/j.joca.2017.08.015

[72] Zhou Y Wang T Hamilton JL et al Wnt/β-catenin signaling in osteoarthritis and in other forms of arthritis. Curr Rheumatol Rep 2017; 19: 53. 28752488 10.1007/s11926-017-0679-z PMC5672801

[73] Kennedy S Ghandehari H Swearingen C et al OP0061 Treatment of knee osteoarthritis with sm04690 improved womac a1 ‘pain on walking’ – results from a 52 week, randomised, double-blind, placebo-controlled, phase 2 study of a novel, intra-articular, Wnt pathway inhibitor. Ann Rheum Dis 2018; 77: 81–82.

[74] Smith MD Triantafillou S Parker A et al Synovial membrane inflammation and cytokine production in patients with early osteoarthritis. J Rheumatol 1997; 24: 365–371. 9034998

[75] Furuzawa-Carballeda J Macip-Rodriguez PM Cabral AR. Osteoarthritis and rheumatoid arthritis pannus have similar qualitative metabolic characteristics and pro-inflammatory cytokine response. Clin Exp Rheumatol 2008; 26: 554–560. 18799084

[76] Goldring MB. Anticytokine therapy for osteoarthritis. Expert Opin Biol Ther 2001; 1: 817–829. 11728217 10.1517/14712598.1.5.817

[77] Caron JP Fernandes JC Martel-Pelletier J et al Chondroprotective effect of intraarticular injections of interleukin-1 receptor antagonist in experimental osteoarthritis. Suppression of collagenase-1 expression. Arthritis Rheum 1996; 39: 1535–1544. 8814066 10.1002/art.1780390914

[78] Pelletier JP Caron JP Evans C et al In vivo suppression of early experimental osteoarthritis by interleukin-1 receptor antagonist using gene therapy. Arthritis Rheum 2005; 40: 1012–1019. 10.1002/art.1780400604 9182910

[79] Fernandes J Tardif G Martel-Pelletier J et al In vivo transfer of interleukin-1 receptor antagonist gene in osteoarthritic rabbit knee joints: prevention of osteoarthritis progression. Am J Pathol 1999; 154: 1159–1169. 10233854 10.1016/S0002-9440(10)65368-0 PMC1866546

[80] Frisbie DD Ghivizzani SC Robbins PD et al Treatment of experimental equine osteoarthritis by in vivo delivery of the equine interleukin-1 receptor antagonist gene. Gene Therapy 2002; 9: 12. 11850718 10.1038/sj.gt.3301608

[81] Chevalier X Goupille P Beaulieu AD et al Intraarticular injection of anakinra in osteoarthritis of the knee: a multicenter, randomized, double-blind, placebo-controlled study. Arthritis Rheum 2009; 61: 344–352. 19248129 10.1002/art.24096

[82] Lacy SE Wu C Ambrosi DJ et al Generation and characterization of ABT-981, a dual variable domain immunoglobulin (DVD-Ig(TM)) molecule that specifically and potently neutralizes both IL-1α and IL-1β. MAbs 2015; 7: 605–619. 25764208 10.1080/19420862.2015.1026501 PMC4622731

[83] Fleischmann R Bliddal H Blanco F et al SAT0575 Safety and efficacy of lutikizumab (ABT-981), an anti–interleukin-1 alpha/beta dual variable domain (DVD) immunoglobulin, in subjects with knee osteoarthritis: results from the randomised, double-blind, placebo-controlled, parallel-group phase 2 trial. Ann Rheum Dis 2018; 77: 1141.

[84] Kloppenburg M Peterfy C Haugen IK et al OP0168 A phase 2a, placebo-controlled, randomized study of ABT-981, an anti-interleukin-1ALPHA and -1BETA dual variable domain immunoglobulin, to treat erosive hand osteoarthritis (EHOA). Ann Rheum Dis 2017; 76: 122. 10.1136/annrheumdis-2018-213336 PMC6390132 30552176

[85] Cheleschi S Cantarini L Pascarelli NA et al Possible chondroprotective effect of canakinumab: an in vitro study on human osteoarthritic chondrocytes. Cytokine 2015; 71: 165–172. 25461395 10.1016/j.cyto.2014.10.023

[86] Ridker PM Everett BM Thuren T et al Antiinflammatory therapy with canakinumab for atherosclerotic disease. N Engl J Med 2017; 377: 1119–1131. 28845751 10.1056/NEJMoa1707914

[87] Schieker M Mindeholm L Praestgaard J et al Interleukin-1β inhibition with canakinumab associates with reduced rates of total hip and knee replacement (THR/TKR) and osteoarthritis (OA) symptoms: exploratory results from the canakinumab anti-inflammatory thrombosis outcomes study (CANTOS). Arthritis Rheumatol 2018; 70(Suppl. 10): abstract 445.

[88] Conaghan PG Bowes MA Kingsbury SA et al Miv-711, a novel Cathepsin K inhibitor demonstrates evidence of osteoarthritis structure modification: results from a 6 month randomized double-blind placebo-controlled phase IIA trial. Arthritis Rheumatol 2017; 69(Suppl. 10): abstract 14L.

[89] Bowes MA Vincent GR Wolstenholme CB et al A novel method for bone area measurement provides new insights into osteoarthritis and its progression. Ann Rheum Dis 2015; 74: 519. 24306109 10.1136/annrheumdis-2013-204052

[90] Conaghan PG Bowes MA Kingsbury SR et al Six months’ treatment with MIV-711, a novel Cathepsin K inhibitor induces osteoarthritis structure modification: results from a randomized double-blind placebo-controlled phase IIA trial. Osteoarthritis Cartilage 2018; 26: S25–S26.

[91] Kingsbury SR Tharmanathan P Arden NK et al Pain reduction with oral methotrexate in knee osteoarthritis, a pragmatic phase III trial of treatment effectiveness (PROMOTE): study protocol for a randomized controlled trial. Trials 2015; 16: 77. 25872649 10.1186/s13063-015-0602-8 PMC4351849

